# Analysis of the Output Characteristics of a Novel Small-Angle Transducer Used in High-Precision Inertial Sensors

**DOI:** 10.3390/s18103478

**Published:** 2018-10-16

**Authors:** Zongyu Chen, Jiuzhi Dong, Xingfei Li

**Affiliations:** 1 State Key Laboratory of Precision Measuring Technology and Instruments, Tianjin University, Tianjin 300072, China; zongyu@tju.edu.cn; 2 Advanced Mechatronics Equipment Technology Tianjin Area Major Laboratory, Tianjin Polytechnic University, Tianjin 300038, China; dongjiuzhi@tjpu.edu.cn

**Keywords:** electromagnetic sensor, inertial sensors, output characteristics, small-angle transducer, rotor with short-circuit ring

## Abstract

This paper presents the design of a novel small-angle transducer characterized by a simple structure, fast response and very low reaction torque. A theoretical model is presented which describes the linear relationship between the output voltage and the angular displacement when the rotor rotates away from the null position. By analysis of the theoretical model, it is revealed that the small-angle transducer possesses a very high linearity within ±4° and a high sensitivity (approximately 0.34 V/°), and the parameters affecting output characteristics can be obtained. Furthermore, it is found that the transducer sensitivity can be improved by optimizing the load impedance and excitation frequency. These findings are verified by numerical evaluations. In addition, the established theoretical model and simulation analysis provide a quantitative method for analyzing the output characteristics of the novel small-angle transducer.

## 1. Introduction

Electromechanical inertial sensors play an important role in inertial navigation and control applications due to their high accuracy and reliability [1,2,3,4,5,6,7,8]. For a given excitation voltage, the angular transducer used in electromechanical inertial sensors is used to provide a voltage output signal that is proportional to the angular displacement of the rotor from a null position [9,10,11,12]. As a critical component of inertial sensors, the requirements for precision from the angular transducer are much higher than those for inertial sensors [13,14,15]. Therefore, the angular transducer is necessary for working within the scope of small angles to provide high sensitivity, linearity, very low reaction torque and high reliability. As microsyn signal generators possess good structural stiffness and high sensitivity [16,17], they are often used in gyroscopes, inertially stabilized platforms and some accelerometers [18,19]. However, the rotor and the stator of a microsyn are both constructed of silicon-steel laminations with high permeability [13]; therefore, a very small non-concentricity between the stator and the rotor leads to the formation of two random reaction torques acting on the output axis: a radial magnetic pull and a tangential electromagnetic reaction torque. It is difficult to compensate for these random reaction torques; thus, it is difficult for a microsyn to realize a very low reaction torque.

In this paper, we present the design of a novel small-angle transducer used in high-precision inertial sensors. Its rotor is a specially shaped conducting loop made from highly conductive and non-conductive magnetic materials. Between the outer stator and the inner stator, which are both constructed of silicon-steel laminations with high permeability, an air gap in the form of a ring emerges, in which the rotor is free to rotate. Moreover, both the excitation windings and the output windings are mounted on the outer stator, so that there are no coil lead wires on the rotor. In addition, because the rotor is made of a highly conductive non-magnetic material (e.g., copper), a radial magnetic pull is not formed on the rotor; therefore, this novel small-angle transducer can achieve a very low reaction torque, so that it is suitable for use in high-precision inertial sensors. Meanwhile, the transducer’s output characteristics directly affect the performance of the inertial sensors. Therefore, it is necessary to have a quantitative method to analyze the output characteristics in order to predict the performance of sensors, and it is also essential to carry out parameter optimization to acquire higher linearity and sensitivity. In addition, the study of the output characteristics can also provide a theoretical basis to further improve precision and reliability and expand the scope of applications.

This paper is organized as follows. In Section 2, the basic structure and working principles are introduced, and the theoretical model that describes the linear relationship between the output voltage and the angular displacement when the rotor rotates away from the null position is deduced through analysis of the magnetic circuit of the transducer. The short-circuit ring on the rotor is regarded as a single-turn short-cut coil and its equivalent magnetic resistance and magneto-reactance are deduced. In Section 3, a simulation tool is introduced, and a simulated analysis is carried out over a range of angles of rotation to acquire the curves of the output voltage. In Section 4, various curves, which are the results of the theoretical model calculation, are discussed and compared with the simulation results, and the parametric optimum design is then realized. It is done through an analysis of the theoretical model over a range of angles of rotation, excitation frequencies and resistances etc. The paper is concluded in Section 5.

## 2. Theory

In this section the working principle of the new small-angle transducer is firstly presented. Secondly, the equivalent magnetic resistance and magnetoreactance of the short-circuit ring (conducting loop) on the rotor is deduced by regarding the short-circuit ring as a single-turn short-cut coil. Thirdly, the theoretical model of the transducer is deduced, while ignoring the magnetic resistance and leakage flux of the stators, to describe the output characteristics of the transducer.

### 2.1. Working Principle 

**Figure 1:**
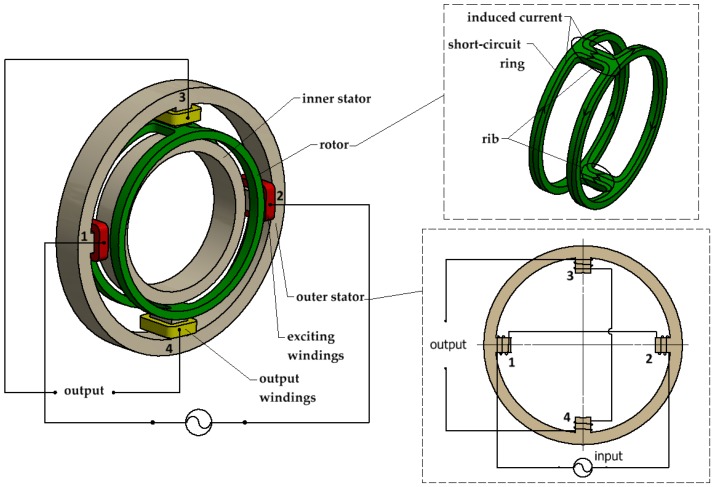
Schematics of the basic structure and windings connection of the transducer.

**Figure 2:**
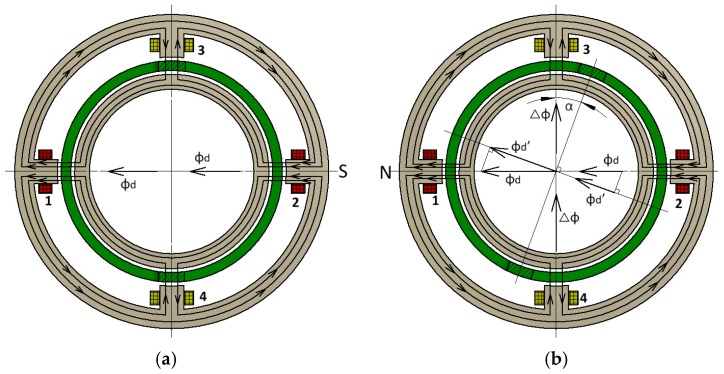
Instantaneous magnetic flux distribution: (**a**) magnetic flux distribution in the null position; (**b**) magnetic flux distribution when the rotor moves clockwise by an angle of α away from the null position.

The basic structure of the transducer is shown in the image on the left in Figure 1. It consists of an outer stator with four salient poles, of which 1 and 2 are excitation poles and 3 and 4 are output poles; an inner stator; and a rotor. More specifically, the rotor is partitioned into two short-circuit rings by two ribs and is free to rotate in the air gap between stators, as shown in the top right-hand image of Figure 1. The excitation windings and output windings are mounted on the excitation poles and output poles, respectively, and are connected in series opposition as shown in the bottom right hand corner of Figure 1. Figure 2 illustrates the instantaneous magnetic flux distribution. It can be seen that an alternating magnetic flux ${\overset{\cdot }{\phi }}_{d}$ (the main magnetic flux) flows through the excitation poles, across the air gap and through the output poles, when an alternating current is applied to the excitation windings. In this paper, the dot symbol indicates a phasor. Moreover, the magnetic flux through the output poles has two parts flowing in and out. The null position of the transducer is shown in Figure 2a. The center of the ribs of the rotor is consistent with the center of the output poles. The magnetic flux flowing in the output pole is equal to the magnetic flux flowing out of the output pole; therefore, the output voltages in the output windings cancel out if the stators’ overlap is symmetrical, the air gaps are uniform, and all coils are identical.

As shown in Figure 2b, if the rotor is rotated clockwise at an angle of α from the null position, the magnetic flux flowing in the short-circuit ring is ${\overset{\cdot }{\phi }}_{d}’$, that is a component of ${\overset{\cdot }{\phi }}_{d}$, and ${\overset{\cdot }{\phi }}_{d}’={\overset{\cdot }{\phi }}_{d}\cdot \cos\alpha$; therefore, the net magnetic flux of each output pole is $\Delta \overset{\cdot }{\phi }$ and $\Delta \overset{\cdot }{\phi }=\frac{1}{2}{\overset{\cdot }{\phi }}_{d}\cdot \sin2\alpha$. When angle α is small, sin2α=2α, so that $\Delta \overset{\cdot }{\phi }={\overset{\cdot }{\phi }}_{d}\cdot \alpha$. Thus, the sum of the voltages in the output windings is no longer zero. Because the output windings are connected in series opposition (shown in the bottom right-hand image of Figure 1), according to the law of electromagnetic induction, ${\overset{\cdot }{E}}_{2}$ can be expressed as

(1) $${\overset{\cdot }{E}}_{2}=2W_{2}\frac{d\Delta \overset{\cdot }{\phi }}{dt}$$

where ${\overset{\cdot }{E}}_{2}$ is the phasor of the induced electromotive force in the output windings, $W_{2}$ is the number of turns of output windings, and $\Delta \overset{\cdot }{\phi }$ is the alternating net magnetic flux of each output pole. Therefore, from Equation (1), it is seen that the output voltage is positively proportional to $\Delta \overset{\cdot }{\phi }$. Consequently, the output voltage is positively proportional to the angular displacement of the rotor from its null position α and thus realizes the measurement of the small angle. Meanwhile, a reversal of the direction of the rotor displacement from the null position will cause a reversal in the polarity of the output voltage.

### 2.2. The Equivalent Magnetic Resistance and Equivalent Magnetoreactance of a Short-Circuit Ring of Rotors

**Figure 3:**
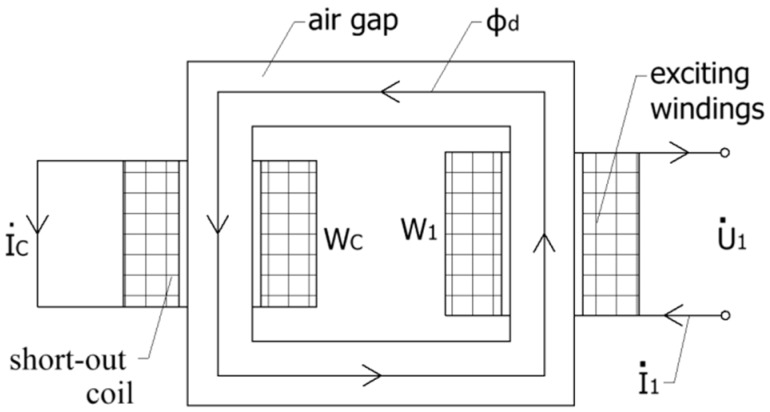
The secondary winding as a single turn short-cut coil.

Based on the working principle of the small-angle transducer, induced eddy currents are generated on the short-circuit ring of the rotor, which consequently generates an obstruction to the main magnetic flux flowing in the short-circuit ring. As shown in Figure 3, the short-circuit ring can be regarded as a single-turn short-cut coil of secondary winding. Therefore, the obstruction to the main magnetic flux can be regarded as the equivalent magnetic resistance and magnetoreactance of the short-circuit ring in the magnetic circuit of the small-angle transducer. Since the coil is a single-turn, the leakage flux of short-cut coil can be ignored, so that we can obtain Equations (2)–(5):

(2) $${\overset{\cdot }{I}}_{1}W_{1}+{\overset{\cdot }{I}}_{c}W_{c}={\overset{\cdot }{\phi }}_{d}\cdot G_{0}$$

(3) $${\overset{\cdot }{E}}_{c}={\overset{\cdot }{I}}_{c}r_{c}$$

(4) $${\overset{\cdot }{I}}_{c}r_{c}=-j\omega W_{c}{\overset{\cdot }{\phi }}_{d}$$

(5) $${\overset{\cdot }{I}}_{1}W_{1}={\overset{\cdot }{\phi }}_{d}\left(G_{0}+j\frac{\omega W_{c}^{2}}{r_{c}}\right)$$

where, ${\overset{\cdot }{I}}_{1}$ is excitation current, ${\overset{\cdot }{I}}_{c}$ is short-cut current in the short-circuit ring, $W_{1}$ is the number of turns of excitation windings, ${\overset{\cdot }{E}}_{c}$ is the induced electromotive force in the short-cut coil, $W_{c}$ is the number of turns of the short-circuit ring ($W_{c}=1$) and $G_{0}$ is the magnetic resistance of the air gap. Therefore, from Equation (5), the equivalent magnetic resistance of the rotor is equal to zero, and the equivalent magnetoreactance of the rotor is equal to $\frac{\omega }{r_{c}}$, where $r_{c}$ is the electrical resistance of one short-circuit ring on the rotor and can be acquired by the dimensions and material of the rotor.

### 2.3. Theoretical Model of Output Characteristics

**Figure 4:**
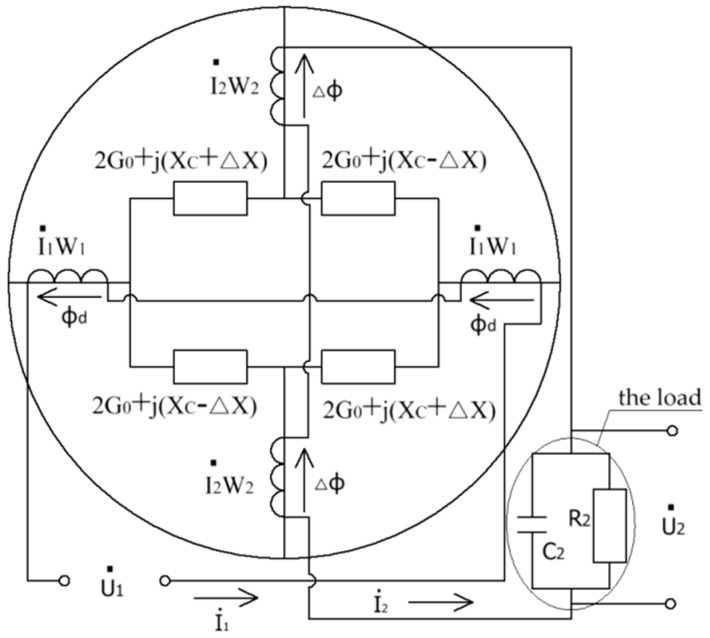
The equivalent electric and magnetic circuits when the rotor rotates clockwise at an angle of α away from the zero position.

Based on the distribution of magnetic flux (Figure 2), we can assume that the magnetic flux flowing through the output poles has two parts flowing in and out; therefore, to perform an in-depth analysis on the magnetic circuit containing the flux, each output pole is divided into two sections from the center. The magnetic flux flows in one section and flows out of the other section. In the zero position, the two magnetic fluxes flowing in and out are in an equal and opposite direction. Consequently, the output voltage in the output windings is zero. Due to the function of the short-circuit ring of the rotor, when the rotor rotates clockwise at an angle of α away from the zero position, the net magnetic flux in the output pole is no longer zero; therefore, the differential flux $\Delta \overset{\cdot }{\phi }$ results from the flux imbalance caused by the displacement from the zero position. Figure 4 shows the equivalent electric and magnetic circuits when the rotor rotates clockwise at an angle of α away from the zero position. By building the electric circuit of the transducer, we can obtain Equations (6)–(8):

(6) $${\overset{\cdot }{U}}_{1}={\overset{\cdot }{I}}_{1}r_{1}-{\overset{\cdot }{E}}_{1}$$

(7) $$-{\overset{\cdot }{E}}_{2}={\overset{\cdot }{I}}_{2}r_{2}+{\overset{\cdot }{U}}_{2}$$

(8) $${\overset{\cdot }{U}}_{2}={\overset{\cdot }{I}}_{2}\cdot \frac{R_{2}}{1+j\omega C_{2}\cdot R_{2}}$$

where ${\overset{\cdot }{U}}_{1}$ and ${\overset{\cdot }{U}}_{2}$ denote the AC excitation voltage and output voltage, respectively, where ${\overset{\cdot }{U}}_{1}=U_{1m}\sin\omega t$, and $U_{1m}$ is the amplitude of the excitation voltage. f is the excitation frequency ($f=\frac{\omega }{2\pi }$), and ${\overset{\cdot }{I}}_{1}$ and ${\overset{\cdot }{I}}_{2}$ denote the current in the excitation windings and output windings, respectively. ${\overset{\cdot }{E}}_{1}$ and ${\overset{\cdot }{E}}_{2}$ denote induced electromotive force in the excitation and output windings, respectively. $r_{1}$ and $r_{2}$ denote resistance of excitation and output windings, respectively. $R_{2}$ is the load resistance, and $C_{2}$ is the load capacitance. By building the magnetic circuit of the transducer, ${\overset{\cdot }{\phi }}_{d}$ and $\Delta \overset{\cdot }{\phi }$ can be written as

(9) $${\overset{\cdot }{\phi }}_{d}=\frac{2{\overset{\cdot }{I}}_{1}W_{1}\left(2G_{0}+jX_{c}\right)-2{\overset{\cdot }{I}}_{2}W_{2}j\Delta X}{4G_{0}{}^{2}+4G_{0}jX_{c}-X_{c}{}^{2}+\Delta X^{2}}$$

(10) $$\Delta \overset{\cdot }{\phi }=\frac{-2{\overset{\cdot }{I}}_{2}W_{2}\left(2G_{0}+jX_{c}\right)-2{\overset{\cdot }{I}}_{1}W_{1}j\Delta X}{4G_{0}{}^{2}+4jX_{c}G_{0}+\Delta X^{2}-X_{c}{}^{2}}$$

where, G0=ln(r+δ)rμ0hθ is the magnetoresistance of the air gap under the single magnetic pole, where r is the diameter of the inner stator, δ is the length of the air gap, h is the thickness of the outer stator, θ is the angular width (in radians) of the magnetic pole and $\mu_{0}$ is the permeability of the vacuum ($\mu_{0}=4\pi \times 10^{-7}\;H/m$). When the structural parameters are confirmed, $G_{0}$ is a constant. $X_{c}$ is the magnetic resistance of the rotor in the zero position ($X_{c}=\frac{\omega }{r_{c}}$), and ΔX is the change in the magnetoreactance of the rotor, when the rotor rotates clockwise at an angle of α away from the zero position. $W_{1}$ and $W_{2}$ denote the turns of excitation windings and output windings, respectively.

Since ${\overset{\cdot }{I}}_{1}\gg {\overset{\cdot }{I}}_{2}$, from Equations (9) and (10),

(11) $$\Delta X=\left(-jG_{0}+\frac{1}{2}X_{c}\right)\cdot \sin2\alpha$$

According to Equations (6)–(11) and the law of electromagnetic induction, ${\overset{\cdot }{U}}_{2}$ can be written as

(12) $${\overset{\cdot }{U}}_{2}=-2j\omega W_{2}\Delta \overset{\cdot }{\phi }=\frac{-4j\omega W_{1}W_{2}Z_{1}{\overset{\cdot }{U}}_{1}R_{2}\left(G_{0}-\frac{1}{2}jX_{c}\right)\sin2\alpha }{Z_{1}Z_{3}\left[r_{2}\left(1+j\omega C_{2}R_{2}\right)+R_{2}\right]+4j\omega W_{2}{}^{2}Z_{3}Z_{2}\left(1+j\omega C_{2}R_{2}\right)}$$

When the angle of rotation α is small, Equation (12) can be written as

(13) $${\overset{\cdot }{U}}_{2}=\frac{-8j\omega W_{1}W_{2}Z_{1}{\overset{\cdot }{U}}_{1}R_{2}\left(G_{0}-\frac{1}{2}jX_{c}\right)\alpha }{Z_{1}Z_{3}\left[r_{2}\left(1+j\omega C_{2}R_{2}\right)+R_{2}\right]+4j\omega W_{2}{}^{2}Z_{3}Z_{2}\left(1+j\omega C_{2}R_{2}\right)}$$

Here, $Z_{1}=4G_{0}{}^{2}+4jX_{c}G_{0}-X_{c}{}^{2}$, $Z_{2}=2G_{0}+jX_{c}$ and $Z_{3}=Z_{1}r_{1}+4j\omega W_{1}^{2}Z_{2}$. Equations (12) and (13) are complex number expressions and comprise real and imaginary components. From Equation (13), it follows that the output voltage ${\overset{\cdot }{U}}_{2}$ is positively proportional to the angle of rotation α and excitation voltage amplitude $U_{1m}$ in the scope of a small angle. Therefore, there is a linear relationship between the output voltage ${\overset{\cdot }{U}}_{2}$ and angle α. Moreover, the output voltage ${\overset{\cdot }{U}}_{2}$ can be given by structural parameters and electromagnetic parameters, as shown in Table 1.

**Figure 5:**
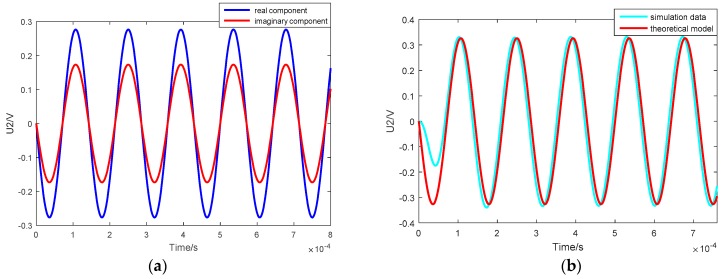
(**a**) The real component and imaginary component of the theoretical model; (**b**) contrast of the curves of output voltage for the simulated and theoretical models.

The result of the real and imaginary components obtained by numerical calculation is shown in Figure 5a, and the curves of the output voltage for the simulation and theoretical models are contrasted in Figure 5b. The fact that the stable curves overlap identically confirms the validity of the theoretical model.

## 3. Simulation

Based on the above theoretical analysis, we can conclude that the output voltage is related to the parameters shown in Table 1. To verify the theoretical model, simulations were carried out over a range of angles of rotation. In addition, the application of the transducer required the dimensions to be in mm. The value of the parameters used in the simulation and theoretical calculation are shown in Table 1.

**Figure 6:**
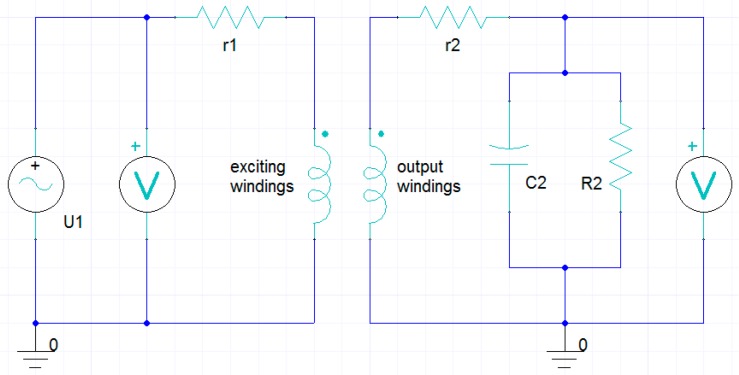
The electric circuit.

The ANSYS Maxwell-3D was used as the simulation tool. It is an interactive software package that uses the finite element method (FEM) and electromagnetic field theory based on Maxwell’s equations. It is often used to simulate and solve three-dimensional electromagnetic field problems of high-precision inertial sensors [20,21]. For transient electromagnetic field, Maxwell’s equations can be rewritten as

(14) $$\left\{ \begin{array}{l} \nabla \times H=\sigma E\\ \nabla \times E=\frac{\partial B}{\partial t}\\ \nabla \times B=0 \end{array}\right.$$

where B is the magnetic flux density, H is the magnetic field intensity, E is the electric field density and σ is the conductivity of the material. First, the magnetic vector potential A (B=∇×A) is introduced for simplified calculation. After substitution, by merger and normalization, the discrete equation of all internal nodes in the computational domain can be deduced. Then, the magnetic flux density B at each time step at every node in the finite element model is obtained. Finally, by importing the electrical circuits connected with the windings shown in Figure 6, the variation curves of the output voltage in the output windings can be obtained.

**Figure 7:**
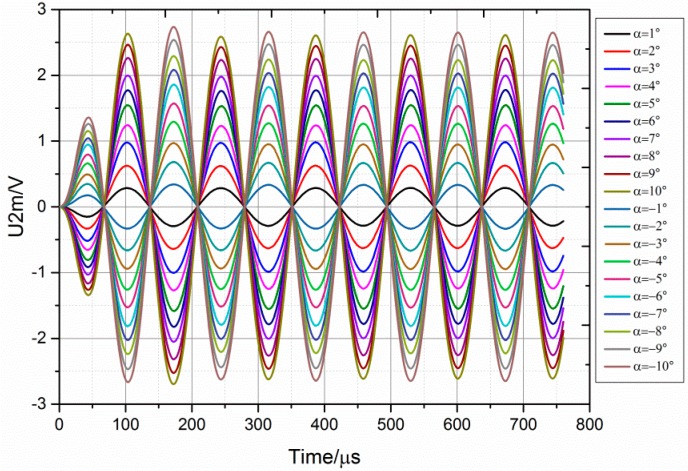
The simulation results of output voltage for the different angles of rotation.

When the rotor rotates clockwise or counterclockwise away from the zero position, the angle of rotation value is defined as positive or negative, respectively. Figure 7 shows the curves for the variation of the output voltage with time when the angle of rotation of the rotor is ±1°, ±2°, ±3°, ±4°, ±5°, ±6°, ±7°, ±8°, ±9° and ±10°. The output voltage amplitude increases with the angle of rotation; however, this trend gradually weakens. In addition, the phases of the curves of the same direction of rotation as the rotor are identical, and the phase difference between the positive angle and negative angle is 180°. Moreover, after a transient period of approximately 300 μs, the stable shape of curves is a standard sine wave. The transient period of approximately 300 μs can be ignored in real engineering applications; hence, we can conclude that the small-angle transducer has a very fast response.

## 4. Results and Discussion

In this section, to verify the influence of the important parameters in the theoretical model, simulations were carried out over a range of angles of rotation, amplitudes of excitation voltage, frequencies of excitation voltage and resistances and capacitances of the load. The linearity and sensitivity of the output characteristics were first analyzed. Then, the results of the theoretical model calculation and simulation were analyzed using various curves of the output voltage amplitude. As a result, by parametric combination, the optimum design was realized.

### 4.1. Analysis of Linearity and Sensitivity 

**Figure 8:**
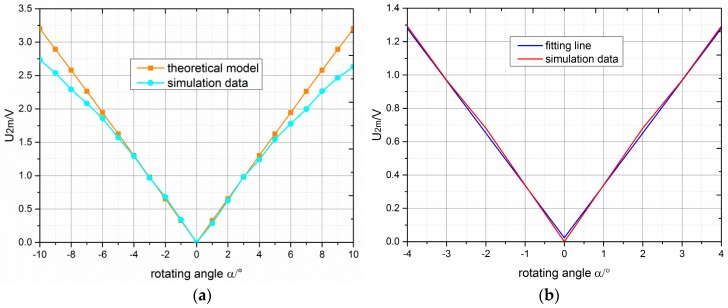
(**a**) Variation of output voltage amplitude with angle of rotation in the theoretical model and simulation; (**b**) linear fitting of simulation data within ±4°.

Linearity and sensitivity are very important parameters in small-angle transducers used in high-precision inertial sensors; therefore, the angle of rotation was varied in the simulation experiment to confirm the scope of the angle in which the transducer possesses a high linearity and sensitivity. As shown in Figure 8a, the variation of the output voltage amplitude $U_{2m}$ with angles of rotation α ranging from −10° to 10° in the theoretical model was compared with that of the simulation results. The curve of the theoretical model was a straight line, and the output voltage amplitude proportionally increased with the absolute value of the angle of rotation. However, the curve of the simulation was no longer a straight line when the absolute value of the angle was greater than 4°. Moreover, the variation trend of the theoretical model agreed with that of the simulation. Meanwhile, the difference between the output voltage amplitude obtained from the simulation and theoretical model increased when the absolute value of the angle of rotation was greater than 4°. The linear fitting graph of the simulation data within ±4° is shown in Figure 8b. The fitting straight-line and curve of simulation data nearly overlapped. We were able to deduce that the transducer possessed a very high linearity within ±4°. Furthermore, when the angle of rotation was ±1°, the output voltage amplitude in both the simulation and theoretical model was about 0.34 V; therefore, the novel small-angle transducer had a very high linearity within ±4° and a high sensitivity (0.34 V/°).

### 4.2. Influence of Parameters on the Amplitude of Output Voltage

Based on the theoretical analysis, we were able to assume that the output voltage amplitude in the theoretical model was positively proportional to the excitation voltage amplitude. In addition, we were able to deduce the influence of the main parameters, i.e., amplitude of excitation voltage, excitation frequency, load resistance and load capacitance, on the output voltage amplitude. Therefore, a simulation of the trend of the variation of the output voltage amplitude with the excitation voltage amplitude ranging from 0 V to 14.14 V was first performed.

**Figure 9:**
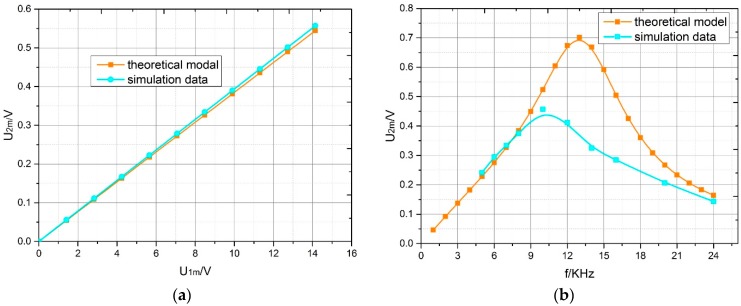
Variation of output voltage amplitude with excitation voltage frequency and amplitude in the theoretical model and simulation; α = 1°: (**a**) excitation voltage amplitude; (**b**) excitation voltage frequency.

For both the theoretical model and simulation results, when the angle of rotation was 1°, the output voltage amplitude increased with the excitation voltage amplitude, and their curves overlapped (Figure 9a). Moreover, we were able to obtain the ratio of the output voltage amplitude and the excitation voltage amplitude was nearly 0.04. To analyze how the output voltage amplitude varied with the three other parameters, the variations of the output voltage amplitude with excitation frequency from 1 KHz to 24 KHz, load resistance from 1 KΩ to 140 KΩ and load capacitance from 0.01 μF to 0.06 μF in the theoretical model were compared with those of the simulation result when the angle of rotation was 1°. In Figure 9b, with an increase in the excitation frequency, the output voltage amplitude first increased and then decreased. The curve of the theoretical model reached its peak at f=14 KHz, and the curve of simulation reached its peak at f=10 KHz. When the excitation frequency was increased to 7 KHz, a larger deviation occurred between the two curves until the excitation frequency reached 21 KHz.

**Figure 10:**
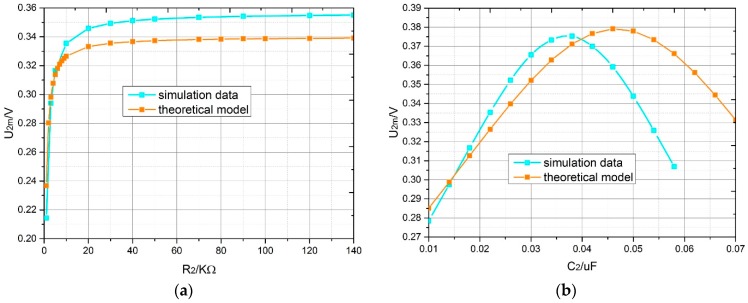
Variation of the output voltage amplitude with the capacitance and resistance of load in the theoretical model and simulation; α = 1°: (**a**) the resistance of load; (**b**) the capacitance of load.

The trend of the output voltage amplitude variation with the load of the theoretical model agreed with that of the simulation (Figure 10). In Figure 10a, with the change of the load resistance, the output voltage amplitude first increased and then tended to be stable from $R_{2}=10\;K\Omega$. Moreover, the difference between the voltages of the theoretical model and the simulation was about 0.01 V. In Figure 10b, with the change of load capacitance the output voltage amplitude first increased and then decreased. Moreover, the output voltage amplitude was highest at $C_{2}=0.034\;\mu F$ and $C_{2}=0.046\;\mu F$ for the simulation and the theoretical model, respectively. The results of the theoretical model were largely consistent with those of the simulation; thus, the theoretical model was verified. The reasons for the deviation were the magnetic saturation of the stator core caused by the excessive excitation frequency, the asymmetry of magnetic field and the performance of the material.

### 4.3. Discussion on the Optimisation of Parameters

From the above analysis, we were able to conclude that the electromagnetic parameters in the theoretical model influenced the output voltage. Moreover, suitably increasing the excitation frequency and load impedance improved sensitivity. Taking into account the influence of the above parameters, the parametric computation for optimal performance was as follows: excitation frequency should be less than 9 KHz, load resistance should be less than 20 KΩ, and load capacitance should be less than 0.04 μF.

## 5. Conclusions

This paper presented a novel small-angle transducer used in high-precision inertial sensors and explored its output characteristics based on theoretical and simulation investigations. The impact of important electromagnetic parameters including excitation voltage and the load impedance were explicitly analyzed, which was useful to improve the performance of the transducer and to expand the scope of its application.

The analysis of this novel transducer demonstrated that it was characterized by a simple structure, fast response and very low reaction torque. Moreover, a theoretical model of the transducer output characteristics was designed to reveal the influences of structural and electromagnetic parameters on the output voltage, providing a method of quantitative analysis. A simulation experiment was carried out to verify the effectiveness of the theoretical model. Analysis of the theoretical model revealed that the transducer possessed a high linearity when the angle of rotation of the rotor was within ±4° and that its sensitivity was approximately 0.34 V/°. Furthermore, the optimum design was realized by parametric combination and the results indicated that the load resistance should be less than 20 KΩ and that the transducer sensitivity could be improved by suitably increasing excitation frequency and load capacitance.

In the future, we will analyze the temperature characteristics and the stability of the zero position of the novel small-angle transducer using theoretical and experimental studies. Extensive field evaluation of the transducer’s performance will also be carried out. Furthermore, further studies should be carried out on the leakage flux and magnetic saturation that occur in stator cores under real working conditions.

## Figures and Tables

**Table 1:** The parameters in the simulation.

Structural Parameters		Electromechanical Parameters	
α	1°	$U_{1m}$ (V)	8.48
r (mm)	26	f (Hz)	7000
δ (mm)	2.3	$r_{1}$ (Ω)	45
h (mm)	5	$r_{2}$ (Ω)	240
θ (rad)	0.21	$C_{2}$ (μF)	0.022
$W_{1}$	200	$R_{2}$ (KΩ)	10
$W_{2}$	560	$r_{c}$ (Ω)	1
